# SOCIAL ENGAGEMENT AND DEPRESSIVE SYMPTOMS: DIFFERENTIAL EFFECTS IN OLDER ADULTS WITH AND WITHOUT CANCER

**DOI:** 10.1093/geroni/igae098.2431

**Published:** 2024-12-31

**Authors:** Natalia Babenko, Joanne Elayoubi, William Haley, Brent Small

**Affiliations:** University of South Florida, New Port Richey, Florida, United States; University of South Florida, Tampa, Florida, United States; University of South Florida, Tampa, Florida, United States; University of North Carolina at Chapel Hill, Chapel Hill, North Carolina, United States

## Abstract

Recent studies have shown that social connection and engagement can serve as protective factors against depression. Buffering effects are sometimes found, suggesting greater protective effects for people experiencing high levels of stress. We examined whether social connection and engagement had a greater protective effect for depressive symptoms in cancer survivors compared to people without cancer. We analyzed longitudinal data from 856 participants with incident cancer and 856 propensity-matched non-cancer controls from the Health Retirement Study. Multilevel modeling was used to examine the associations between social connection and engagement and changes in depressive symptoms over time. Social connection and engagement were generally associated with fewer depressive symptoms before and after the onset of cancer (or a matched timepoint for controls). These protective effects were stronger for cancer survivors who were partnered and lived in a larger household compared to controls. Participants with cancer who were partnered pre-cancer experienced a significant decrease in depressive symptoms after cancer compared to partnered controls who did not exhibit decline. Becoming partnered in post-cancer period led to decreased depressive symptoms compared to the pre-cancer partnership status in both groups. Consistent with the buffering hypothesis, results suggest that social connection is generally advantageous, especially for people with cancer in a post-cancer period. Prospective public health interventions increasing social engagement to build resilience in the overall population may enhance psychological resilience and facilitate recovery from cancer. Future research should examine whether people with cancer benefit from support-enhancing interventions and whether this effect also occurs in other illness populations.

